# Relationship between Psychological Factors and Social Support after Lifting of Evacuation Order in Fukushima Prefecture, Japan

**DOI:** 10.3390/bs10100149

**Published:** 2020-09-29

**Authors:** Yujiro Kuroda, Yohei Koyama

**Affiliations:** 1 Research Department, Fukushima Prefectural Centre for Environmental Creation, Fukushima 960-7700, Japan; 2 Center for Integrated Science and Humanities, Fukushima Medical University, Fukushima 960-1295, Japan; ykoyama@fmu.ac.jp

**Keywords:** social support, psychological/behavioral medicine, professional support, disaster public health, social determinants of health

## Abstract

We examined the relationships among social support and psychological variables and investigated the status of social support among villagers whose evacuation order had been lifted. A written questionnaire was posted to 4828 registered residents of Iitate Village; 1405 valid responses were received. The main finding (in joint assessment by local and external experts) was the “need for professional support” (191 respondents, 13.6%). A multivariate analysis found that among those living in permanent housing outside the village, the need for support was significantly more likely for those without emotional support or instrumental support than for those not providing support. The associations between perceived social support and living environment suggest the need to strengthen social support measures in areas where evacuation orders are yet to be lifted, and provide useful information for examining the effects of future support efforts.

## 1. Introduction

After the accident at the Fukushima Daiichi Nuclear Power Station in March 2011, as one of protective measures against exposure to radiation, the Japanese government issued mandatory evacuation orders for residents living in the areas affected by radioactive fallout [1]. As of 2020, the evacuation orders in most areas have been lifted, and some residents have returned to their hometowns. The situations have changed as time proceeded and the measures of radiation protection need to be elaborated [2]. However, radiation was not the only problem for the residents. In the short term, problems arose from evacuation and relocation, due mainly to shortages of human and material resources and infrastructure (e.g., hospital ward closures) [3,4]. In the medium term, evacuation has resulted in lifestyle changes, stress, and post-traumatic stress disorder, with consequential lifestyle diseases and depression [5,6]. In the long term, a transformation of society and culture (community issues), and changes to the nation’s energy policies can be expected [7,8,9]. Although radiation was no doubt a critical risk factor in the nuclear accident aftermath, the affected population has also suffered secondary health problems and living difficulties due to the evacuation and lifestyle changes. In this paper, we focus on the social impact of the nuclear accident on residents of Iitate Village and discuss potential roles of social supports for their psychological states.

Iitate Village is a farming village with a population of around 6000, located in the Hamadori region of Fukushima Prefecture. Located 30 to 50 km from the Fukushima Daiichi Nuclear Power Plant, it was not evacuated immediately after the accident, but instead received evacuees from coastal areas. However, owing to the spread of radioactive material, the entire village became contaminated and was designated as a planned evacuation zone on 11 April 2011 [10]. Collectively, the villagers were forced to evacuate to prefabricated temporary housing units in the prefecture or rent an apartment on their own within and outside the prefecture. Although they did not have to pay the rent in either case, their life as evacuees became long term, continuing until March 2017.

Previous research has indicated that long-term evacuees experienced multiple stressors, including uncertainty about the future, lifestyle changes, and psychological stress, which have resulted in deterioration of both physical and mental health [11,12,13,14]. Analysis of the relationship between the evacuees’ living environment and their levels of mental health and social support indicated that those residing in rental apartments had poorer mental health and more difficulty getting social support than those residing in temporary housing [8]. These results have been shared with Iitate Village’s health authorities, and projects designed to strengthen the social ties among residents, such as a “Pop-Up Cafe Enterprise”, have been undertaken in areas with high populations of evacuees [7]. Although the villagers have now been allowed to resume their lives, their lifestyles have diversified, with some moving back to their village homes, some having made new lives for themselves in their new towns, and others still living in temporary housing. At the time of the survey described here, only 259 residents (5%) had returned to Iitate Village, a lower rate than in other areas where evacuation orders have been lifted. In interviews, the villagers frequently spoke of the loss of social ties [15]. Thus, the social ties that were developed during the villagers’ long-term evacuation were once again severed with the lifting of evacuation orders, raising concerns about the worsening of physical and mental health.

Social ties are important in earthquake disaster recovery [16,17,18]. For example, in the aftermath of the 1994 Great Hanshin Awaji Earthquake, social capital was the most influential factor in the residents’ recovery, even when earthquake damage, regional characteristics, and economic circumstances were taken into consideration [16]. Research on the role of social ties in recovery from the Great East Japan Earthquake disaster has centered on the tsunami-affected regions of Iwate and Miyagi Prefectures. Research focusing on the nuclear disaster has been limited to studies which we have conducted. Residents of Miyagi Prefecture who were evacuated as a group, experienced better preservation of social capital than evacuees who were relocated to individual rental apartments [19]; moreover, low social capital was related to poorer mental health [20]. Therefore, rebuilding social ties is a pressing issue in regions where the community has been disbanded at some stage because of forced evacuation.

Several studies have shown long-term effects on mental health after the Great East Japan Earthquake [5,6], but their results have not necessarily been applied to public health activities. For example, subjects who scored poorly on a mental health survey may have already had social support, which did not fit the conventional correlation between mental health and social support that has been widely assumed by many public health experts. However, their approach often overlooked that the social condition, mental health, and social support that were specific to the local community were not necessarily correlated. For this reason, it was important that we involved local health professionals, often who were public health nurses in local government, in the assessments of the health survey and mobilized their local knowledge for the assessment. They were responsible for providing local people with health advice and welfare services, and they were at the forefront of risk communication with them. More importantly, they were closely involved in the life of the local people, and they belonged to the same community, making them mediators between local people and external experts. Joint assessments of the survey results by local and external experts would enable greater practical application.

The main aims of this study were to examine the relationship between social support and psychological factors, and to investigate the status of social support among villagers whose evacuation order had been lifted, by using joint assessment by local and external experts, among returnees to Iitate Village and evacuees still living outside the village. A previous study by Murakami et al. reported that the well-being of returnees was higher than that of non-returnees, but the authors’ intensive fieldwork in Iitate Village for many years suggested otherwise. Regardless of whether they returned or not, they faced psychological problems, and the loss of their sense of belonging to a community and loss of the community itself seemed to be major causes for those problems. Thus, the practical hypothesis of this study was that both groups faced psychological health problems which were related to their social connections. Different types of social connections (emotional or instructional) had different impacts. Clarifying the relationship between their psychological state and each type of social connection can lead to better measures for evacuees. This study was not necessarily theory-based, and we did not have a clear theoretical hypothesis, but it was to meet the rather urgent and practical needs in the field.

## 2. Methods

### 2.1. Participants

This study was a collaboration between Fukushima Medical University and the Iitate Village Municipal Government. In April 2017, a written questionnaire was posted to 4828 registered residents of the village aged 18 years or older and living within the Prefecture, with a notification of a general health check-up. Responses were collected over a 10-day period in May 2017 at the check-up, or by return post. The check-up was carried out in Iitate Village and in four evacuation areas (Fukushima City, Date City, Soma City, and Minamisoma City). Of the 1415 completed questionnaires, 10 were excluded because the main outcome variable (i.e., need for professional support) was missing. Data from 1405 participants were included in the final analysis.

This survey was conducted as part of a health program within the health and welfare section of the Iitate Village government. Before a health examination, local government officials explained the nature of the survey and obtained written consent. The Ethics Committee of Fukushima Medical University granted its consent (no. 2609).

### 2.2. Survey Items

#### 2.2.1. Main Outcome Variable

The main outcome was the presence or absence of residents’ ”need for professional support”. We examined the content of questionnaire responses (see variables in the Section 2.2.2 and Section 2.2.3) to understand the state of their psychological stress, and whether or not their perceived social support (PSS) was adequate [21]. The determination of each resident’s need for professional support was a multilayered process, which was unique to this study. There were four evaluation criteria as follows: (1) content of questionnaire responses; (2) interview content (clinical information obtained by local health professionals); (3) history of previous support; and (4) other background information (from local experts with long-term relationships with residents, including whether they had support before the disaster, their family structure, and their relationships with the community). Since the survey was conducted one year after the long-term evacuation order was lifted, their living environment was likely to change. In the interview, local health professionals asked them about their health and also about their daily lives. After obtaining the first and second evaluation criteria, i.e., (1) and (2), we discussed it with three public health nurses from Iitate Village and one public health nurse from Fukushima Prefecture, who were familiar with the residents, to understand evaluation criteria (3) and (4). Thus, we made an overall evaluation on each case and determined whether or not each resident needed professional support.

After the survey, as part of the health program of the health and welfare section of Iitate Village government, we provided the residents who needed professional support with the support from a psychiatrist or clinical psychologist.

#### 2.2.2. Main Explanatory Variables

The main explanatory variable was perceived social support (PSS). Generally, PSS is broadly categorized as “receiving support” and “providing support”. In this study, “receiving support” was assessed using the five PSS items proposed by Muraoka [22], which were comprised of two items on “emotional support” (someone to talk to when having difficulties and someone to talk to when feeling unwell) and three items on “instrumental support” (someone to help with activities of daily living, someone to take one to the doctor, and someone to provide personal care). Providing support’ was assessed with the widely used item “listening to worries and concerns” [23]. Items were answered as “present/absent”.

#### 2.2.3. Related Factors

The investigation items used, in this study, were decided upon after discussion with village public health nurses. Information was gathered on (1) basic characteristics (age, gender, current family structure, and presence/absence of co-morbidities); (2) living environment (residing within the village, in temporary housing outside the village, or in permanent housing outside the village); and (3) psychological variables (simplified Japanese version of the Athens Insomnia Scale [AIS] and presence/absence of stress).

The main living environments at the time of the survey were comprised of the following: “residing within the village” (persons who had returned to Iitate Village, were registered as residents, and spent most of their time there); “residing in temporary housing outside the village” (evacuees who spent most of their time living in temporarily rented housing outside Iitate Village); and “residing in permanent housing outside the village” (evacuees who spent most of their time living permanently in new homes outside Iitate Village).

The simplified Japanese version of the AIS consists of eight sleep-related items (sleep quality, sleep-onset insomnia, night awakening, early morning awakening, insufficient sleep, decreased daytime well-being, reduced daytime physical/mental activity, and daytime drowsiness) rated as “present” (1 point) or “absent” (0 points), and the total score was calculated as the sum of item scores [24].

### 2.3. Statistical Analysis

First, participants were sorted into two groups based on their need for professional support, and descriptive statistics for basic characteristics, living environment, PSS, and psychological variables were calculated (Table 1). Means and standard deviations were calculated for continuous variables, and a Student’s *t*-test was used to assess differences between the groups. Frequency and percentage were used for categorical variables, and differences were tested by a Chi-squared test. Subsequently, for each living environment (i.e., residing within the village, in temporary housing outside the village, or in permanent housing outside the village), PSS types (i.e., emotional support, instrumental support, and providing support) were set as explanatory variables, and the presence/absence of need for professional support was set as the outcome variable, then logistic regression analysis was carried out. The odds ratios (ORs) and 95% confidence intervals (CIs) were determined for these relationships (Table 2). In addition, items with a significant difference in Table 1 (namely, gender, family structure, AIS scores, sleep score, and presence/absence of stress) were input as moderator variables in a binomial logistic regression analysis, and adjusted ORs (AORs) and 95% CIs were calculated. The level of significance for all analyses was set at *p* < 0.01. All statistical analyses were conducted with IBM SPSS Statistics for Macintosh (version 24.0, IBM Corp., Armonk, NY, USA).

## 3. Results

A total of 191 (13.6%) of the 1405 respondents indicated signs that they might have needed professional support (Table 1). By living environment, 27 out of 216 (12.5%) residents residing within the village, 74 out of 509 (14.5%) in temporary housing outside the village, and 86 out of 624 (13.8%) in permanent housing outside the village needed professional support, with no significant differences. The need for professional support was significantly associated with being female, living alone, absence of social support on all six PSS items, high sleep score (poor sleep status), and presence of stress.

The associations between PSS and the need for professional support in each living environment (Table 2) indicated that village residents without emotional support or instrumental support were significantly more likely to need professional support than those who had such support. Residents of temporary housing outside the village without instrumental support were significantly more likely to need it. Residents in permanent housing outside the village without emotional support or instrumental support or who did not provide support were significantly more likely to need it.

Multivariate analysis using moderator variables found no significant association between PSS and continued support among villagers residing within the village or in temporary housing outside the village. However, among those residing in permanent housing outside the village, those without emotional support (AOR = 2.87, 95% CI = 1.52–5.42) or instrumental support (AOR = 1.86, 95% CI = 0.99–3.48), or not providing support (AOR = 1.87, 95% CI = 1.05–3.36) were significantly more likely to need professional support.

Multivariate analysis including the AIS score as a moderator variable showed that those with poor sleep status were significantly more likely to need professional support than those with good sleep status across all living environments (within the village, AOR = 1.50, 95% CI = 1.18–1.91; temporary housing outside the village, AOR = 1.42, 95% CI = 1.24–1.64; permanent housing outside the village, 1.50, 95% CI = 1.30–1.74).

## 4. Discussion

### 4.1. Relationship between PSS and Need for Professional Support

We investigated the relationship between the need for professional (psychosocial) support and the PSS status of long-term evacuees from the Fukushima nuclear disaster in relation to their living environment following the lifting of the evacuation order.

The need for professional support did not differ between the three living environments considered. This suggests that village returnees, residents remaining in temporary housing, and those deciding not to return each had their own psychosocial health issues that required professional follow-up. Of particular interest were the results of stratified analysis by living environment, which indicated a significant relationship between the need for professional support, and receipt of emotional support, receipt of instrumental support, and provision of support among residents in permanent housing outside of the village. Note that our survey was undertaken in the first year after the evacuation order was lifted, when many residents were still in temporary housing. At that time, many residents were still living in the temporary housing units where the community still functioned, and therefore their social relationships with one another were partially maintained. Prior research has illustrated the importance of both receiving and providing emotional support for maintaining good health [23]. It is arguable that the presence of the functioning community in the temporary housing units could have provided them with opportunities to receive and provide social support for one another. For example, in the temporary housing units, they were likely to be given social roles in various residents’ associations and collective activities.

Meanwhile, there were people who had already made permanent housing arrangements outside the village and decided not to return to the village. While losing the belongingness to the original community, many of them failed to build their belongingness to the new community. The tendency of being unable to gain the belongingness to neither of those communities, hence towards isolation, seemed more apparent, in this group. It might have severely restricted their opportunities to receive or provide support, as is evident in our results. Therefore, being cut off from their original community may have increased their need for emotional support.

In terms of strategic measures, there is a need for activities to increase evacuee PSS such as on-going provision of information using village magazines and increasing support through cooperation with local authorities in evacuee areas. It is also important to provide opportunities for evacuees living outside the village to comfortably gather together. The Iitate Village residents’ association of Minamisoma holds a monthly” small tea ceremony” for evacuees who have built new houses in Minamisoma. Undertaking on-going efforts such as these and developing a framework to deploy them is important.

### 4.2. Relationship between Sleep Status and Need for Professional Support

Sleep disorders tended to be common among evacuees dealing with changes in their living environment [25]. Our findings similarly indicated a significant association between sleep disturbances and the need for professional support. A comparison with other conditions that could be revealed by clinical test results showed that the treatment of disaster-related sleep disorders tends to be delayed. However, as sleep disorders are known to increase the risk of circulatory and cardiovascular disorders and hypertension [26], the importance of dealing with such conditions needs to be shared with support teams. Sleep disorders exhibit a range of patterns such as sleep-onset insomnia and night awakening. Recent research indicates that it is possible to reduce these symptoms by incorporating cognitive behavioral therapy into sleep hygiene education rather than just relying on sleeping medications [27,28].

The findings of this study resulted, in part, from assessments conducted in collaboration with both local and external experts in a local clinical practice setting. Specifically, all subjects who needed support were visited by local public health nurses, and when more specialized support was needed, psychiatrists from the research team visited to provide advice and referrals to local medical institutions. Such efforts should contribute to the education of local public health nurses who must deal with future long-term mental health problems and is an important example of desirable professional relationships.

## 5. Conclusions

This study has several limitations. First, residents evacuated to various locations outside the Prefecture did not participate. It cannot be ruled out that the social factors emphasized here may be worse for such evacuees. In addition, it was not possible to determine causal relationships because of the cross-sectional nature of our data. While longitudinal research including evacuees from outside the prefecture may become necessary, the strength of this study is in the clarification of “current” issues, which can inform specific measures.

As the provision of temporary housing was scheduled to finish at the end of March 2019, residents were confronted with the decision whether or not to return to the village. Regardless of their choice, professional supports must be made available to all residents. Needless to say, they experienced a great deal of hardship after the nuclear accident. The characteristics of the association between PSS and living environment, as revealed here, suggest the necessity of strengthening the social support to maintain and improve psychological status of residents even in areas where evacuation orders have not been lifted. In addition, this study provided useful information with which to examine the effect of future support efforts. The insights can also be used in times of other disasters that may require different forms of evacuations, and they can be used as a reference for supporting residents to adapt to new lifestyles.

## Figures and Tables

**Table 1:** Relationships among need for professional support and study variables.

Variables	Need for Prof. Support (*n* = 191)	No Need for Prof. Support (*n* = 1214)	*p*-Value
**Basic characteristics**			
Age (elderly)	114 (59.7)	704 (58.3)	0.752
Gender (female)	127 (66.5)	652 (54.0)	0.001
Current family structure (living alone)	36 (19.1)	124 (10.3)	0.001
Co-morbidities (present)	67 (45.0)	464 (41.5)	0.428
**Living environment**			
Residing within the village	27 (14.4)	189 (16.3)	0.766
Residing in temporary housing outside the village	74 (39.6)	435 (37.4)	
Residing in permanent housing outside the village	86 (46.0)	538 (46.3)	
**Perceived social support (PSS)**			
Someone to talk to when having difficulties (absent)	59 (30.9)	132 (10.9)	<0.001
Someone to talk to when feeling unwell (absent)	48 (25.1)	92 (7.6)	<0.001
Someone to help with daily life activities (absent)	66 (34.6)	191 (15.8)	<0.001
Someone to take one to the doctor (absent)	42 (22.0)	94 (7.8)	<0.001
Someone to provide personal care (absent)	51 (26.7)	143 (11.8)	<0.001
Listening to worries and concerns (absent)	74 (38.9)	280 (23.2)	<0.001
**Psychological variables**			
Sleep score (mean ± SD)	4.6 ± 2.0	2.7 ± 2.1	<0.001
Stress (present)	167 (87.9)	816 (67.5)	<0.001

**Table 2:** Relationships among PSS and need for professional support in different living environments.

		Residing within the Village (*n* = 216)		
Type of PSS		Need for Prof. Support (*n* = 27)	OR (95% CI)	AOR (95% CI)
Emotional support	Present (*n* = 181)	17 (9.4)	1.00	1.00
	Absent (*n* = 35)	10 (28.6)	3.86 (1.59–9.37)	1.76 (0.50–6.14)
Instrumental support	Present (*n* = 167)	16 (9.6)	1.00	1.00
	Absent (*n* = 49)	11 (22.4)	2.73 (1.17–6.37)	1.81 (0.52–6.31)
Providing support	Present (*n* = 159)	16 (10.1)	1.00	1.00
	Absent (*n* = 56)	11 (19.6)	2.19 (0.95–5.05)	1.63 (0.54–4.98)
		**Residing in Temporary Housing Outside the Village** **(*n* = 509)**		
**Type of PSS**		**Need for Prof. Support (*n* = 74)**	**OR (95% CI)**	**AOR (95% CI)**
Emotional support	Present (*n* = 448)	57 (12.7)	1.00	1.00
	Absent (*n* = 60)	17 (28.3)	1.88 (0.89–3.98)	2.02 (0.89–4.59)
Instrumental support	Present (*n* = 415)	50 (12.0)	1.00	1.00
	Absent (*n* = 93)	24 (25.8)	1.98 (1.05–3.75)	1.87 (0.93–3.76)
Providing support	Present (*n* = 370)	48 (13.0)	1.00	1.00
	Absent (*n* = 138)	26 (18.8)	1.02 (0.56–1.87)	1.05 (0.55–2.00)
		**Residing in permanent housing outside the village (*n* = 624)**		
**Type of PSS**		**Need for prof. support (*n* = 86)**	**OR (95% CI)**	**AOR (95% CI)**
Emotional support	Present (*n* = 536)	54 (10.1)	1.00	1.00
	Absent (*n* = 87)	32 (36.8)	3.20 (1.77–5.77)	2.87 (1.52–5.42)
Instrumental support	Present (*n* = 520)	56 (10.8)	1.00	1.00
	Absent (*n* = 103)	30 (29.1)	1.94 (1.08–3.47)	1.86 (0.99–3.48)
Providing support	Present (*n* = 477)	49 (10.3)	1.00	1.00
	Absent (*n* = 145)	36 (24.8)	1.86 (1.10–3.16)	1.87 (1.05–3.36)

Adjustment variables included gender, age, family structure, total sleep score, and stress. OR, odds ratio; AOR, adjusted odds ratio; CI, confidence interval.
